# Correlations between Obstructive Sleep Apnea Syndrome and Periodontitis: A Systematic Review and Meta-Analysis

**DOI:** 10.3390/dj12080236

**Published:** 2024-07-26

**Authors:** Marco Portelli, Ignazio Russo, Angela Mirea Bellocchio, Angela Militi, Riccardo Nucera

**Affiliations:** Department of Biomedical and Dental Sciences and Morphofunctional Imaging, University of Messina, 98125 Messina, Italy; ignazio.russo@studenti.unime.it (I.R.); angelamirea@live.it (A.M.B.); amiliti@unime.it (A.M.); riccardo.nucera@unime.it (R.N.)

**Keywords:** OSAS, periodontitis, inflammation, oxidative stress, xerostomia

## Abstract

The focus of this article was to evaluate the link between obstructive sleep apnea syndrome (OSAS) and periodontitis, considering various hypotheses supporting the relationship between respiratory disorders and periodontitis. The literature review for this study was performed using the PubMed, Google Scholar, Cochrane library, and Proquest databases. The review process was guided by the PRISMA guidelines. The PECOS protocol (Population, Exposure, Control, Outcome, Study) was followed in developing the search strategy to ensure consistent and accurate selection of articles. To evaluate quality, cross-sectional studies were reviewed using the Joanna Briggs Institute (JBI) critical appraisal tool. Case-control studies were assessed with the Newcastle–Ottawa Scale (NOS). The research included a total of 10 studies, encompassing 88,040 participants. The meta-analysis observed a statistically significant association between OSAS and periodontitis, with an odds ratio OR = 2.4620 (95%-CI: 1.7345–3.4946 *p* ≤ 0.0001). The results suggest a potential association between OSA and periodontitis. Further investigations are warranted to confirm this association and elucidate its underlying mechanism.

## 1. Introduction

Obstructive Sleep Apnea Syndrome (OSAS) is a respiratory condition marked by recurrent instances of full (apnea) or partial (hypopnea) blockage of the upper air passages during sleep. OSAS affects nearly a billion patients aged between 30 and 69 years [1]. Clinical manifestations of OSAS include snoring, disturbances of concentration, excessive daytime sleepiness, and cognitive decline, often associated with reduced blood oxygen saturation [2]. Polysomnography is the gold standard for OSAS diagnosis; it is a sleep-time study evaluating heart rate, breathing, eye movements, brain and muscular activity during sleep. OSAS is classified based on the number of apnea–hypopnea episodes observed during one hour of sleep: Apnea–Hypopnea Index (AHI). Intermittent hypoxia leads to oxidative stress, resulting in systemic dysfunction. Untreated obstructive sleep apnea syndrome (OSAS) in fact is associated with vascular conditions (atherosclerosis, heart failure, and coronary heart disease) and impaired glucose tolerance, insulin resistance, and diabetes [3,4]. Intermittent hypoxia induces an excess production of reactive oxygen species (ROS) [5], causing cellular damage and activating several inflammatory signaling pathways, including the proinflammatory nuclear factor kappa B (NF-kB), which leads to leukocyte activation and release of inflammatory cytokines [6]. Periodontitis is a chronic inflammatory disease involving the destruction of the tooth-surrounding tissue. It produces a loss of clinical attachment (CAL), alveolar bone loss (ABL), presence of periodontal pockets, and probing bleeding [7,8]. Periodontitis is related to plaque dysbiosis in patients with an individual susceptibility; the accumulation of biofilm promotes the development of bacterial species such as Fusobacterium nucleatum, responsible for a gingival inflammatory response and the release of ammonia, which promotes the proliferation of bacteria like Porphyromonas gingivalis [8]. It can result in an exaggerated inflammatory response with excessive release of cytokines [9], reactive oxygen species (oxidative stress), and matrix metalloproteinases, causing damage to periodontal tissues. Therefore, periodontitis is a persistent inflammatory disease that is non-communicable and chronic, with 80% of periodontal damage caused by the host response [10]. Severe periodontitis affects an estimated 1.1 billion individuals globally, as reported by Global Burden of Disease data [11]. The inflammation of periodontal tissues produces effects also at a systemic level; it pockets ulcerated epithelium, allows blood dissemination of bacteria, and leads tocytokines spreading through the circulatory system [12]. Periodontitis is often associated with multiple systemic diseases, including diabetes [13,14,15], cardiovascular diseases [15,16,17], and respiratory disorders [15]. Patients affected by moderate to severe OSAS are characterized by a prevalence of periodontitis [18,19]. During sleep, apnea and hypopnea states caused by OSAS lead to oxidative stress, and the reactive oxygen species produced are responsible for an inflammatory process. Indeed, individuals with OSAS tend to exhibit increased levels of interleukin 6 (IL 6) [20], elevated serum concentrations of high-sensitivity C-reactive protein (hs-CRP), and gingival crevicular fluid interleukin-1β [19]. IL6 and IL-1β are cytokines implicated in periodontal damage. Additionally, OSAS induces a stress state, which represents a risk factor for periodontitis [21]. Patients with OSAS tend to breathe through their mouths during sleep in an attempt to compensate for the obstruction caused by partial or total collapse of the upper airways. Oral respiration produces xerostomia, and the reduced cleansing action of the saliva results in plaque accumulation; all this triggers an inflammatory process that gradually leads to the development of periodontitis. OSAS and periodontitis share several risk factors such as obesity, diabetes, alcohol consumption, and smoking. These common risk factors contribute to the development and exacerbation of both conditions. The focus of this study is to evaluate the link between OSAS and periodontitis in studies that performed OSAS diagnosis with polysomnography evaluation.

## 2. Materials and Methods

### 2.1. Protocol

We conducted this systematic review following the PRISMA statement and adhering to the recommendations of the Cochrane Handbook for Systematic Reviews of Interventions (version 5.1.0) [22]. This review plan received the ID: CRD42024529836 upon its registration in the PROSPERO.

### 2.2. Research Strategy and Information Sources

The present study employed a systematic literature review using several prominent databases: PubMed, Google Scholar, Cochrane Library, and Proquest. The search strategy adhered to the PECOS protocol (Population, Exposure, Control, Outcome, Study) to ensure methodological rigor and consistency in article selection.

### 2.3. Eligibility Criteria

Inclusions criteria (PECOS):

- Participants:
  1. Observational studies exploring the relationship between OSAS and periodontitis.
  2. Study population comprising subjects of both sexes.
  3. Inclusion of studies with available data regarding the diagnosis of OSAS and periodontitis.
- Exposure:
  1. Diagnosis of obstructive sleep apnea confirmed by polysomnography.
- OSAS severity categorized according to standardized criteria, the apnea–hypopnea index (AHI).
- Comparison:
  Control group not diagnosed with OSAS.
- Outcomes:

Presence and severity of periodontitis (diagnosis based on clinical examination of periodontal tissues and assessment of periodontal parameters).

- Studies were eligible if they employed cross-sectional, cohort, or case-control designs, with a minimum sample size of 15 subjects.
- Exclusion criteria:
  1. Studies comprising case series, case reports, or cohorts involving fewer than 15 patients.
  2. Books, abstracts, and editorials.

Four electronic databases were used to search for studies that met the inclusion criteria:

1. PubMed,
2. Google scholar,
3. Cochrane library,
4. Proquest.

These databases were searched for studies published until March 2024. During the study selection process, the exclusion of duplicate articles was conducted. This ensured the removal of duplicates, guaranteeing that each study was considered only once in the analysis. We conducted our search with a focus on studies reported in English and limited it to those that included human subjects. The following search strategy was applied: “((OSA) OR (OSAS)) AND (PERIODONTAL DISEASE)”.

### 2.4. Data Extraction

Two reviewers conducted independent data extraction for the study characteristics; any disagreements had been resolved by discussion with another author. Data extraction was performed independently by the two reviewers using a predesigned Excel extraction sheet with the following items: authors, year, study design, country, periodontitis definition, OSAS definition, cases, controls. All disagreements were addressed through consultation with another author.

### 2.5. Quality Assessment

For the quality assessment of case-control studies (Table S2), it was used the Newcastle–Ottawa Scale (NOS) [23]. The quality assessment of cross-sectional studies was performed using the Joanna Briggs Institute (JBI) critical appraisal tool (Table S1) [24].

### 2.6. Statistical Analysis

Data relating to the diagnosis of OSAS and periodontitis were extracted from individual studies, and a meta-analysis was performed using the Mantel–Haenszel method for the common-effect model and the inverse variance method for the random-effects model. Heterogeneity was assessed using the Q test and I2.

Sensitivity tests were conducted to examine the effect of individual studies on overall heterogeneity. Potential bias was assessed using funnel plot analysis (Figure S1), test for funnel plot asymmetry, and linear regression test of funnel plot asymmetry. The analysis was performed through the “R studio” statistic software (2023 ver.12.1 + 402 program).

## 3. Results

### 3.1. Search Results and Articles Selection

**Figure 1:**
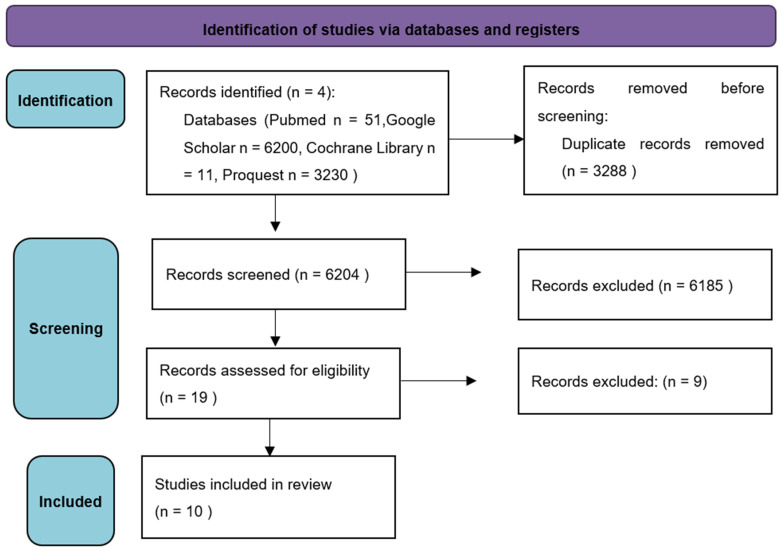
Flow chart of study selection performed according tot PRISMA guidelines.

The flowchart summarizing the selection process is depicted in Figure 1. The search yielded the following: 51 titles on PubMed, 6200 on Google Scholar, 11 on Cochrane library, and 3230 on Proquest registers. Electronic searches have identified 9492 studies. Of these, 6204 articles remained after the duplicates removal and were examined on the basis of title and abstract. After this first screening, 6185 articles were excluded based on the title and abstract text, and the remaining 19 were examined for eligibility based on the full text. At the end of this further selection 10 studies were identified.

### 3.2. Results

**Figure 2:**
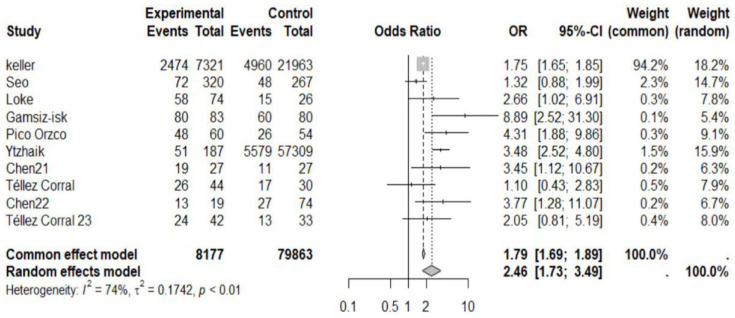
Forest plot of the association between OSAS and periodontitis [18,19,25,26,27,28,29,30,31,32].

The meta-analysis included a total of 10 studies (Table 1): 8 cross-sectional studies and 2 case-control studies, involving a total of 88,040 participants. We observed a significant association between OSAS and periodontitis, with an odds ratio (OR) of 2.4620 (95%-CI: 1.7345–3.4946, *p* ≤ 0.0001) (Figure 2). No significant publication bias was observed (Linear regression test of funnel plot asymmetry = *p*-value = 0.1274. Test for funnel plot asymmetry = *p* = 0.0799). The sensitivity analysis indicated that the exclusion of a single study did not result in significant changes to the estimates.

Nine of the ten included studies [18,19,25,26,28,29,30,31,32] showed an association between OSAS and periodontitis, except [27], which found an association between AHI and plaque percentage. Study [26] found an association between OSAS and probing depth (PD) and clinical attachment loss (CAL). The authors [31] found a prevalence of stage 3 periodontitis in severe OSAS patients, suggesting a relationship between OSAS severity and periodontitis. In line with this, Pico-Orozco et al. [18] found a significant correlation between the apnea–hypopnea index (AHI) and probing depth (PD), and between AHI and clinical attachment loss (CAL). Additionally, article [27] found an association between AHI and plaque percentage. Study [32] found higher levels of bleeding on probing (BOP) and CAL in OSAS patients, and also found an association between low oxygen saturation levels and periodontitis, suggesting the latter as a predictive factor. However, the same study [32] found a weak correlation between OSAS severity and periodontitis. Conversely, The research in [25] did not find a significant correlation between periodontal indices and OSAS. Study [19], despite finding an association between OSAS and periodontitis, did not find significant differences in the association between periodontitis and mild OSAS and periodontitis and moderate/severe OSAS. Additionally, ref. [25] did not find gender differences in the association between OSAS and periodontitis. Two other articles observed a variation in plaque composition with a prevalence of Candida [31] or Prevotella [28]. The studies [19,30] found, in addition to an association between the two diseases, higher levels of inflammation. The authors of [30] found increased levels of interleukins 6, 17, and 33 in saliva, while interleukins 17 (IL-17) and 33 (IL-33) were found in gingival crevicular fluid (GCF). The study [19] found elevated levels of interleukins 1β (IL-1β) in gingival crevicular fluid (GCF) and high-sensitivity C-reactive protein (hs-CRP).

## 4. Discussion

This meta-analysis evaluates the link between OSA and periodontitis. This review is, to our knowledge, the first to include only studies that employed polysomnography to diagnose OSAS. A total of 10 studies and 88,040 participants were selected for the quantitative and qualitative analysis. The review has led to a discovery: a notable association between obstructive sleep apnea syndrome (OSAS) and periodontitis. The observed odds ratio (OR = 2.4620, 95%-CI:1.7345–3.4946 *p* ≤ 0.0001) underscores the strength of this connection. It is pertinent to acknowledge that the actual relationship between OSAS and periodontitis needs to be demonstrated. The connection between the two conditions is hypothesized to be linked to four aspects: oxidative stress, inflammation, oral breathing, and the presence of common risk factors. The alternation of hypoxia and reoxygenation states that the occurrence in patients with OSAS is responsible for the production of reactive oxygen species and leads to oxidative stress, that lead to a chronic inflammatory state. This mechanism could explain why individuals with OSAS tend to exhibit increased levels of IL6 and elevated serum concentrations of high-sensitivity C-reactive protein (hs-CRP) and IL-1β in crevicular fluid (GCF). Since IL6 and IL-1β are cytokines implicated in periodontal damage, this observation could explain not only the association between the two conditions, but also the direct proportionality between the degree of OSAS and the severity of inflammation and consequently of periodontal damage, in accordance with the study [15], where a statistically significant correlation was found between AHI and PD and CAL. OSAS may sustain plaque dysbiosis over time, and this occurs through the inflammatory process that causes the release of collagen proteins and apoptotic cells in crevicular fluid, allowing the development of periodontopathogenic bacteria. Furthermore, patients with OSAS tend to breathe through the mouth during sleep in an attempt to compensate for upper airway obstruction caused by partial or total collapse of the upper airways. Oral breathing leads to xerostomia [33], reducing the cleansing action of saliva, and this produce the accumulation of dysbiotic plaque. Recent studies have observed changes in plaque composition in individuals with OSAS [34], particularly an increase in Prevotella [28] and *Cutibacterium* spp. [35], which seem to be implicated in gingivitis, although their role is not yet well defined. OSAS patients develop high levels of mental and physical stress, as a consequence of poor rest during sleep [21]. Stress represents a risk factor for periodontitis [36]. OSAS and periodontitis share several other common risk factors such as obesity, diabetes, alcohol consumption, and smoking. Smoking not only intensifies the inflammatory response within the periodontium but also accentuates the oxidative stress associated with OSAS, thus amplifying the risk of periodontal disease progression. Studies have demonstrated a significant impact of smoking on periodontal health, highlighting its role in increasing periodontal inflammation and tissue destruction, and also in determining changes in the microbiome [37]. Furthermore, smoking has been implicated in the pathogenesis of OSAS, exacerbating upper airway inflammation; the study [38] showed an association between smoking and chronic pharyngitis in OSAS patients. Therefore, the synergistic effect of smoking in conjunction with OSAS underscores the importance of smoking cessation interventions as a crucial component of both periodontitis and OSAS management strategies [39]. Addressing smoking cessation alongside OSAS treatment may lead to improved therapeutic outcomes and mitigate the risk of periodontal complications associated with both conditions. Obesity represents a significant shared risk factor that exacerbates the interaction between OSAS and periodontitis. Studies have clarified the complex relationship between obesity and both OSAS and periodontal disease, further emphasizing their inter-connection and clinical implications. Research [40,41] has highlighted the association between obesity and OSAS, emphasizing obesity as a predisposing factor for the development and progression of OSAS. Obesity contributes to upper airway narrowing and accumulation of adipose tissue around the neck, predisposing individuals to airway obstruction during sleep. Obesity has been implicated also in periodontal disease pathogenesis, through various mechanisms. Adipose tissue secretes pro-inflammatory cytokines such as tumor necrosis factor-alpha (TNF-α) and interleukin-6 (IL-6), which can exacerbate periodontal inflammation and tissue destruction. Furthermore, obesity is often associated with insulin resistance and dyslipidemia, which further contribute to systemic inflammation and compromise periodontal healing processes. Moreover, studies [42,43] have demonstrated a positive correlation between obesity and periodontitis severity, with obese individuals exhibiting a higher prevalence and extent of periodontal disease compared to non-obese individuals and representing an independent predictor of a less favorable response to nonsurgical periodontal treatment. Alcohol consumption represents another significant shared risk factor contributing to the complex interaction between OSAS and periodontitis. Studies have highlighted the harmful effects of alcohol on both conditions, elucidating its role in exacerbating pathogenesis and progression. Studies [44,45] have highlighted the association between alcohol consumption and OSAS, emphasizing the potential for alcohol to exacerbate airway collapse during sleep and worsen the severity of OSAS symptoms. An association was found between alcohol consumption and periodontitis. It should be noted that this result is congruent with previous meta-analyses [46,47]; however, what distinguishes this study is the exclusive inclusion of research diagnosing OSAS by polysomnography. This underscores the robustness of the results and the importance of adopting an accurate diagnostic method such as polysomnography for future research on this relationship. The other reviews [46,47,48], however, included studies that did not use polysomnography but relied on questionnaires [49,50,51] or home sleeping monitors [52]. Although all valid approaches, polysomnography represents the gold standard [53,54]. In review [48], one study was included [55] that classified periodontal patients according to Armitage’s (1999) classification, which we believe is obsolete. No previous review has attempted to explore the mechanisms through which OSA might support plaque dysbiosis. Instead, we believe that future research should focus on plaque modifications in OSAS patients. According to the authors of the article [56,57], systemic inflammation (potentially driven by oxidative stress [6]) could progressively lead to plaque dysbiosis.

### Limitations

This meta-analysis presents some limitations, first of all that in the included studies, OSAS and periodontal health assessment had been conducted using different parameters. Regarding the assessment of OSAS, although all patients underwent polysomnography, there are studies [25,31] where patients are distinguished into OSAS and non-OSAS, but without specifying a reference AHI value. In other studies [28], the sample was divided based on the apnea–hypopnea index (AHI), with AHI <5 considered as non-OSAS and AHI > 5 as OSAS. In study [26], patients are distinguished into non-OSAS (AHI < 5/h), mild moderate OSAS patients (AHI 5–10/h), and severe OSAS (AHI > 10/h). Some studies [32] have classified OSAS as mild (AHI 5–10/h), moderate (AHI 10–15/h), and severe (AHI > 15/h). In other studies [18,19,27,29,31], patients with OSAS are divided into three subgroups: mild (AHI 5–14/h), moderate (AHI 15–29/h), and severe (AHI ≥ 30/h). In study [29], OSAS is defined as a patient with AHI > 15/h of sleep or between AHI 5 and 14/h plus associated symptoms such as sleepiness, fatigue, or insomnia. These classification differences have not allowed a clear definition of the link between the severity of OSA and periodontitis, which could be the subject of future studies. In particular, in light of possible treatment, research should focus on randomized controlled trials to evaluate whether treatment of OSAS would improve periodontal parameters.

Regarding the assessment of periodontitis, the literature lacks uniformity in disease classification. Despite the potential variability of parameters, all studies adhered to the most recent diagnostic criteria for periodontitis, assessing clinical attachment level (CAL) and radiographic bone loss [58], while avoiding reliance on Armitage et al. (1999) classification or probing depth. Most studies did not rely on the current classification criterion for periodontitis [7], but rather utilized the AAP/CDC classification. This classification allows defining a case of periodontitis based on the loss of interdental attachment and probing depth, recognizing four forms of severity: No periodontitis, no evidence of mild, moderate, or severe periodontitis. Mild periodontitis: ≥2 interdental sites with CAL ≥ 3 mm and ≥2 interdental sites with PD ≥ 4 mm (not on the same tooth) or a site with PD ≥ 5 mm. Moderate periodontitis: ≥2 interdental sites with CAL ≥ 4 mm (not on the same tooth) or ≥2 interdental sites with PD ≥ 5 mm (not on the same tooth). Severe periodontitis: ≥2 interdental sites with CAL ≥ 6 mm (not on the same tooth) and ≥1 interdental site with PD ≥ 5 mm. This classification was accepted in our inclusion criteria as it is very similar to the current definition of periodontitis. Today, indeed, a case of periodontitis is defined when there is loss of interdental attachment in at least two non-adjacent sites or vestibular/lingual attachment loss equal to 3 mm with a probing depth less than 4 mm, always in at least two non-adjacent sites to exclude local factors (fractures, cervical caries, incongruous restorations). The only study that does not explicitly refer to CAL was [25]; the periodontal parameters in that case are probing depth (PD) and radiographic bone loss (ABL), and the study was included because, in accordance with [58], to define a case of periodontitis it is sufficient to observe bone loss on radiography, even with orthopantomographic examination. Although the included studies did not use the same evaluation parameters, representing a limitation to our study, all studies that carried out polysomnography and periodontal evaluation in accordance with the most recent provisions of the American Academy of Periodontology (AAP) and the European Federation of Periodontology (EFP) were included.

## 5. Conclusions

From the results obtained in the present meta-analysis, it is possible to state that there is a relationship between OSAS and periodontitis (OR = 2.4620, 95%-CI: 1.7345–3.4946 *p* ≤ 0.0001); however, it could neither be concluded whether there is a relationship between the severity of OSAS and periodontitis, nor whether there is a relationship between the apnea–hypopnea index (AHI) and periodontal indices. Further studies are needed to explain the mechanisms through which these two diseases are related; we suggest investigating changes in the oral microbiome in OSAS patients. Research should evaluate whether treatment can improve periodontal health in order to optimize diagnostic and therapeutic approaches. OSAS patients should have their periodontal health status closely monitored to early-intercept any state of distress of the tooth’s supporting tissues. Additionally, it is essential for these patients to control risk factors and adopt a healthy lifestyle to minimize possible complications, including periodontal disease.

## Figures and Tables

**Table 1:** Characteristics of included studies.

Study	Design	Sample	OSAS Variables	Periodontal Variables	Results
Keller et al. Taiwan (2013) [25]	Cross-sectional	Total N = 114	PSG	PD, ABL	There is an association between OSAS and a prior diagnosis of periodontitis because of the higher prevalence in cases (33.8) compared to controls (22).
Seo et al. Kores (2013) [26]	Cross-sectional	Total N = 687	AHI < 5/h (No OSAS) AHI = 5–10/h (Mild OSAS) AHI > 10/h (Severe OSAS)	PD, CAL	The 60 of those diagnosed with periodontitis also had OSA. OSA was found to be positively associated with periodontitis, probing pocket depth and clinical attachment loss (CAL) in a dose-response manner.
Loke et al. U.S.A (2015) [27]	Cross-sectional	Total N = 100	AHI 5–14/h (Mild), AHI 15–29/h (Moderate), AHI > 30/h (Severe)	REC, BOP, PI, PD, CAL	Significant association was found between apnea–hypopnea index and plaque, but not with periodontitis.
Gamsiz-Isik et al. Turkey (2017) [19]	Case control	Total N = 163	AHI 5–14/h (Mild), AHI 15–29/h (Moderate), AHI 30/h (Severe)	PI, GI, BOP, PD, CAL, % Sites with PD = 4 mm	There was a higher prevalence of periodontitis and higher levels of GCF IL-1 and serum hs-CRP in OSAS patients.
Pico Orozco et al. Spain (2020) [18]	Case control	Total N = 114	AHI 5–14/h (mild), AHI 15–29/h (moderate), and AHI > 30/h (severe)	PI, GI, BOP, PD, CAL, calculus index	The prevalence of periodontitis was higher in subjects with OSAS compared to the controls, and AHI was correlated with PD and CAL.
Chen et al. China (2021) [28]	Cross-sectional	Total N = 54	AHI < 5/h (No OSAS) AHI > 5/h (OSAS)	PD, BOP(%), CAL	A significant association was observed between obstructive sleep apnea and periodontitis. The regression analysis identified the lowest oxygen saturation to be significantly associated with the prevalence of periodontitis.
Ytzhaik et al. Israel (2022) [29]	Cross-sectional	Total N = 132.529	(1) AHI 5–14/h (2) AHI > 15/h	PD, CAL, ABL	OSAS is linked to dental morbidity, particularly periodontitis.
Téllez Corral et al. Colombia (2022) [30]	Cross-sectional	Total N = 93	AHI 5–14/h (Mild), AHI 15–29/h (Moderate), AHI > 30/h (Severe)	PD, CAL, BOP(%), PI, %Sites PD > 4 mm	There is association with stage 3 and OSAS, particularly prevalent in men.
Téllez Corral et al. Colombia (2023) [31]	Cross-sectional	Total N = 75	PSG	PD, CAL, BOP, PI	The cryptic microorganisms within the oral microbiota of patients with periodontitis and OSAS are present as potential pathogens.
Chen et al. China (2023) [32]	Cross-sectional	Total N = 93	AHI = 5–10/h (Mild OSAS) AHI > 10/h (Severe OSAS)	PD, CAL	The structure of the salivary microbial community was altered in patients with OSAS, with an increase in Prevotella. This could explain the high prevalence of periodontitis in OSAS patients.

AHI: Apnea–Hypopnea Index; PSG: Polysomnography; BOP: Bleeding on Probing; CAL: Clinical Attachment Loss; PD: Probing Depth; PI: Plaque Index; GI: Gingival Index; REC: Recession; ABL: Alveolar Bone Loss.

## Data Availability

All the data are reported in the present manuscript.

## Supplementary Materials

The following supporting information can be downloaded at: https://www.mdpi.com/article/10.3390/dj12080236/s1, Figure S1. Funnel plot for the association between OSA and Periodontitis; Table S1. Quality assessment of included cross-sectional studies (Joanna Briggs Institute; JBI); Table S2. Quality assessment of included case-control studies (Newcastle–Ottawa scale; NOS).
