# Supplementary material for: Correlations between Obstructive Sleep Apnea Syndrome and Periodontitis: A Systematic Review and Meta-Analysis
Source: Dent J (Basel). 2024 Jul 26;12(8):236. doi: 10.3390/dj12080236 (PMC11352505; doi:10.3390/dj12080236)
Supplement: Supplementary file 1 [file dentistry-12-00236-s001.zip › dentistry-3075508-supplementary.pdf]

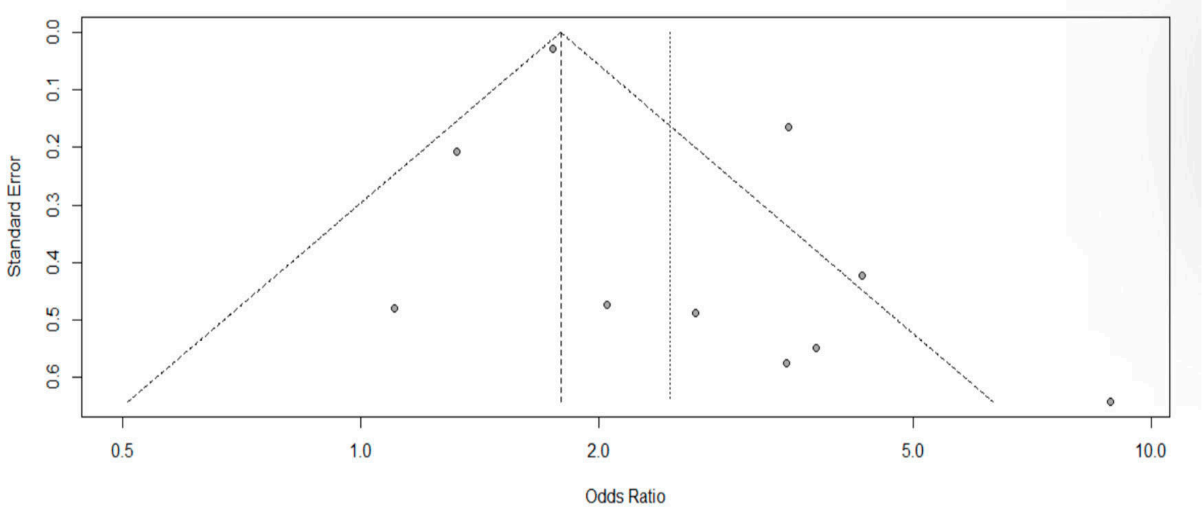

Figure S1. Funnel plot for the association between OSA and Periodontitis

Table S1. Quality assessment of included cross-sectional studies (Joanna Briggs Institute;JBI)

Study	Inclusion criteria clearly defined	Study Subjects and settings described in detail	valid and reliable exposure measurement	Standard criteria for condition measurement	Confoudin g factors identified	Manageme ntof confoundin g factors	Valid reliableoutc ome measuremen t	appropriat e statistical analysis	overall risk
keller et al (2013)	1/1	1/1		1/1	1/1	1/1	1/1	1/1	7/8
loke et al (2015)	1/1	1/1	1/1	1/1	1/1	1/1	1/1	1/1	8/8
seo et al (2013)	1/1	1/1	1/1	1/1	1/1	1/1	1/1	1/1	8/8
Chen et al (2021)	1/1	1/1	1/1	1/1			1/1	1/1	6/8
Ytzhaik et al 2023	1/1	1/1	1/1	1/1			1/1	1/1	6/8
Tellez Corral et al (2022)	1/1	1/1	1/1	1/1			1/1	1/1	6/8
chen et al (2023)	1/1	1/1	1/1	1/1	1/1	1/1	1/1	1/1	8/8
tellez corral et al (2023)	1/1	1/1	1/1	1/1			1/1	1/1	6/8

Table S2. Quality assessment of included case-control studies (New castle-ottawa scale;NOS)

**STUDY**		**SELECTION**			**COMPARABILITY**		**OUTCOME**		**TOTAL**
	ADEQUATE CASE DEFINITION	REPRESENTATIVENESS OF THE CASES	SELECTION OF CONTROLS	DEFINITION OF CONTROLS	COMPARANILITY OF CASES/CONTROLS (MAX 2 STARS)	ASSESMENT OF EXPOSURE	ASCERTAINMENT FOR CASES/CONTROLS	NON-RESPONSE RATE	
PICO ORZCO et al (2021)	1/1	1/1	1/1	1/1	1/2	1/1	1/1		7/9
GAMSIZ –ISIK et al 2017	1/1	1/1	1/1	1/1	1/2	1/1	1/1		7/9
